# Transcriptome analysis reveals nuclear-encoded proteins for the maintenance of temporary plastids in the dinoflagellate *Dinophysis acuminata*

**DOI:** 10.1186/1471-2164-11-366

**Published:** 2010-06-10

**Authors:** Jennifer H Wisecaver, Jeremiah D Hackett

**Affiliations:** 1 Department of Ecology and Evolutionary Biology, University of Arizona, P.O. Box 210088, Tucson, AZ 85721, USA

## Abstract

**Background:**

*Dinophysis *is exceptional among dinoflagellates, possessing plastids derived from cryptophyte algae. Although *Dinophysis *can be maintained in pure culture for several months, the genus is mixotrophic and needs to feed either to acquire plastids (a process known as kleptoplastidy) or obtain growth factors necessary for plastid maintenance. *Dinophysis *does not feed directly on cryptophyte algae, but rather on a ciliate (*Myrionecta rubra*) that has consumed the cryptophytes and retained their plastids. Despite the apparent absence of cryptophyte nuclear genes required for plastid function, *Dinophysis *can retain cryptophyte plastids for months without feeding.

**Results:**

To determine if this dinoflagellate has nuclear-encoded genes for plastid function, we sequenced cDNA from *Dinophysis acuminata*, its ciliate prey *M. rubra*, and the cryptophyte source of the plastid *Geminigera cryophila*. We identified five proteins complete with plastid-targeting peptides encoded in the nuclear genome of *D. acuminata *that function in photosystem stabilization and metabolite transport. Phylogenetic analyses show that the genes are derived from multiple algal sources indicating some were acquired through horizontal gene transfer.

**Conclusions:**

These findings suggest that *D. acuminata *has some functional control of its plastid, and may be able to extend the useful life of the plastid by replacing damaged transporters and protecting components of the photosystem from stress. However, the dearth of plastid-related genes compared to other fully phototrophic algae suggests that *D. acuminata *does not have the nuclear repertoire necessary to maintain the plastid permanently.

## Background

Endosymbiosis, the process through which a once free-living organism becomes an organelle, is a major driver of eukaryotic evolution, enabling hosts to acquire novel characteristics. An excellent example of this process is plastid endosymbiosis, which has distributed photosynthesis across diverse eukaryotic lineages [1]. The primary plastids of the Archaeplastida (green, red, and glaucophyte algae) arose through an endosymbiotic relationship between a heterotrophic eukaryotic host and cyanobacteria [2]. Through subsequent plastid acquisitions, the plastids of both green and red algae were spread to other eukaryotes (e.g., chromalveolates, euglenoids, chlorarachniophytes). Most plastids are long-established organelles, resulting from ancient events and are drastically different from their free-living ancestors, having lost or transferred most genes to the host nucleus [3,4]. One theory of plastid acquisition outlines several key steps in this transition to permanent organelle [5-7]. First, a specific relationship develops between endosymbiont and host. Most hypothetical examples of this process evoke a predator-prey relationship such as a phagotrophic eukaryote continually feeding on algae. The second step is the establishment of a mechanism for controlled metabolic exchange. Lastly, the endosymbiont is reduced to an organelle through gene loss and gene transfer to the host nucleus. In most permanent plastids, these steps were accomplished long ago leaving little clues as to the mechanisms and timing of these events.

The discovery of several organisms that have undergone more recent endosymbioses may provide insights into the first crucial steps of this process. The testate amoeba *Paulinella chromatophora *has a novel primary plastid derived from a *Synechococcus*-like cyanobacterium [8,9]. The endosymbiont genome has already been reduced compared to free-living cyanobacteria, but not as much as the primary plastids of the Archaeplastida [10]. There are also several examples of more recent endosymbioses in the dinoflagellates. Whereas most photosynthetic dinoflagellates have a plastid containing the photopigment peridinin, some have replaced this plastid with one acquired from haptophytes, diatoms or green algae [11]. In these organisms, the early stages of endosymbiosis have been completed and the plastids are permanent organelles.

Plastid retention from prey, also known as kleptoplastidy, is an example of a specific relationship between two organisms that could represent an early stage of plastid acquisition. The organelle is not yet under the complete control of the host and these relationships could serve as a model for understanding the early stages of endosymbiosis in microbial eukaryotes [11-13]. Plastid retention is a form of mixotrophy whereby a feeding cell temporarily sequesters the plastids of prey in order to benefit from the photosynthesis occurring in the stolen organelle. These transient plastids, called kleptoplasts, are found in many eukaryotic lineages including dinoflagellates, ciliates, other unicellular eukaryotes, and even sea slugs [14-17]. These organisms must reacquire their stolen plastids, presumably because they lack necessary nuclear-encoded genes required for plastid maintenance and replication. Most kleptoplastidic organisms can maintain their temporary plastid for several days, but some, such as dinoflagellates of the genus *Dinophysis *maintain their plastids for months through unidentified mechanisms [18,19].

**Figure 1:**
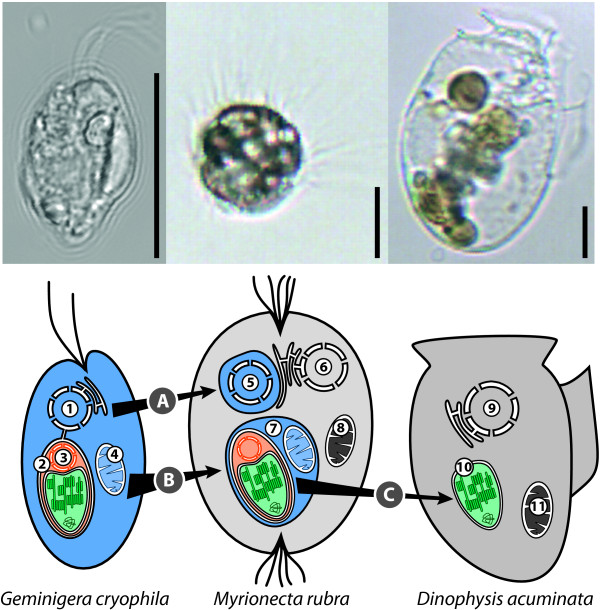
**Kleptoplast acquisition in *M. rubra *and *D. acuminata***. The cryptophyte nucleus (A) and complete cryptophyte plastid and mitochondria (B) are retained in *M. rubra*. When the plastid is acquired by *D. acuminata *the outer two membranes and nucleomorph are lost (C). 1, cryptophyte nucleus; 2, plastid; 3, nucleomorph; 4, cryptophyte mitochondrion; 5, cryptophyte nucleus and cytoplasm surrounded by host membrane; 6, ciliate nucleus; 7, plastid-mitochondrial complex surrounded by host membrane; 8, ciliate mitochondrion; 9, dinoflagellate nucleus; 10, kleptoplast; 11, dinoflagellate mitochondrion. Light photomicrographs of the cells are shown above the cartoon for each organism (scale bar = 10 μm).

Plastids derived from the *Geminigera/Teleaulax *species cluster of cryptophytes have been identified in two different microbial eukaryotes, the ciliate *Myrionecta *and the dinoflagellate *Dinophysis*. Molecular evidence suggests that these ciliates and dinoflagellates temporarily acquire their plastids through plastid retention. Co-isolated species of *Geminigera*, *Myrionecta*, and *Dinophysis *have been shown to have identical 16S plastid gene sequences [20,21] and are distinguishable from other co-isolated strains from different geographic localities [22]. However, contrary to the molecular evidence, the modifications to plastid ultrastructure in both the ciliate and dinoflagellate, compared to the original plastid in *Geminigera *are suggestive of permanent plastid modifications (Figure 1). In the cryptophyte, the plastid is surrounded by four membranes and contains a centrally located pyrenoid [22]. In addition, the plastid includes a nucleomorph, a remnant red algal nuclear genome that encodes an additional 30 genes required for plastid function [23]. When *Myrionecta *consumes the cryptophyte, the mitochondria and complete plastid, including the nucleomorph, are retained [24]. *Myrionecta *separately sequesters the cryptophyte nucleus and expression of plastid genes from the captured nucleus and nucleomorph has been demonstrated [25]. This ability to replenish plastid proteins as they age may explain why the organelles remain active for more than 10 weeks in the ciliate. *Dinophysis *feeds on *Myrionecta rubra*, but there is disagreement as to whether these algae feed to acquire new plastids or simply growth factors needed to maintain the organelle [22]. The plastids found in *Dinophysis acuminata *are composed of only the inner two membranes and the plastid genome, and the cryptophyte nucleus and nucleomorph are absent [26]. Additionally, the pyrenoid is terminally located and the plastids are clustered together forming a compound stellate structure. Despite lacking the cryptophyte nucleus and nucleomorph, *Dinophysis *is able to maintain the plastid for a similar length of time as *M. rubra *[19].

There is some debate as to whether the plastids in *D. acuminata *are kleptoplasts or permanent plastids. The dinoflagellate must feed on *M. rubra *to be grown in laboratory culture, but it has not been definitively demonstrated that the purpose of feeding is to acquire physical plastids. We sequenced the transcriptomes of *D. acuminata*, *M. rubra *and *G. cryophila *to determine if *Dinophysis *contains nuclear-encoded genes that aid in the maintenance of its plastid. These data were analyzed for the presence of plastid genes and were examined for their evolutionary origins and plastid targeting peptides.

## Results and Discussion

### Transcriptome sequencing

We synthesized poly(A) primed cDNA using RNA extracted from cultures of *D. acuminata *taking advantage of the trans-spliced leader sequence present on mature dinoflagellate transcripts. Dinoflagellate transcripts are modified in vivo by the addition of the identical 22 bp trans-spliced leader sequences to the 5' end of all mRNAs [27,28]. Use of this dinoflagellate-specific leader sequence as a 5' primer site during the cDNA amplification step significantly biased the *D. acuminata *cDNA pool toward full-length, nuclear-encoded, dinoflagellate transcripts. The cDNA was randomly sheared and sequenced using 454 FLX Titanium pyrosequencing. Sequencing produced 10.8 megabases of data that assembled into 5,991 unique contigs. There are no sequenced dinoflagellate genomes available to aid in estimating the number of genes in *D. acuminata*, however, transcriptional profiling of the dinoflagellate *Alexandrium tamarense *identified 30,917 unique gene signatures, suggesting that only a fraction of the total transcriptome of *D. acuminata *was sequenced [29]. From the finished assembly, 816 contigs were fully annotated by Blast2GO, and 16 contigs were identified as potentially plastid-related (i.e., cellular compartment GOslim term of plastid or thylakoid, see additional File 1). Of the 16 candidate contigs, three were determined to be nuclear-encoded, plastid-targeted genes: a photosystem II subunit (*psb*U), plastid ferredoxin (*pet*F), and a gene encoding an auxiliary light-harvesting protein (LI818). The remaining 12 contigs were either plastid-encoded and introduced into the cDNA pool through mispriming of the oligo dT primer (photosystem I subunit E), or only peripherally related to plastid function (e.g., sec61 protein translocator). Two additional contigs, a second photosytem II gene (*psb*M) and a plastid phosphate transporter (TPT), were identified as plastid-targeted through sequence similarity searches but were not annotated by Blast2GO because of their high e-value scores. Full-length cDNA sequences complete with the dinoflagellate-specific, trans-spliced leader motif, 5' untranslated region (UTR), and 3' UTR were obtained by PCR from *D. acuminata *for all five genes and used for subsequent phylogenetic analyses and targeting peptide predictions (Table 1).

**Table 1:** Nuclear-encoded plastid proteins of *D. acuminata*

Accession	Annotation	454 (bp)	mRNA (bp)	Phylogenetic grouping
HM125143	Photosystem II subunit M, PsbM	506	506	Cryptophytes
HM125145	Triose-phosphate transporter, TPT	406	1434	Peridinin dinoflagellates
HM125141	Plastid ferredoxin, PetF	155	754	Peridinin dinoflagellates
HM125142	Light harvesting protein LI818	911	1493	Fucoxanthin dinoflagellates
HM125144	Photosystem II subunit U, PsbU	689	938	Haptophytes

In addition, we synthesized *G. cryophila *and *M. rubra *poly(A) primed cDNA that was sequenced by the same method, and the data assembled into 17,997 and 27,723 contigs, respectively. These contig numbers are likely overestimates of the transcriptome sizes of these organisms because multiple contigs can represent a single transcript due to gaps in the assembly. However, the 17,997 contigs are consistent with *G. cryophila *having a gene number similar to other sequenced unicellular algae, which have 5,000-15,000 genes [30]. Likewise, the *M. rubra *contig number is also in agreement with gene numbers from the sequenced ciliate genomes [31,32]. BLASTN comparisons of the three assemblies showed that none of the nuclear-encoded genes in *G. cryophila *or *M. rubra *matched those in *D. acuminata *at the nucleotide level, indicating that the *D. acuminata *dataset is not contaminated with ciliate or cryptophyte nuclear transcripts. Cryptophyte homologs of all the nuclear-encoded, plastid-related genes of *D. acuminata *were identified using BLASTX, with the exception of ferredoxin because it is plastid encoded in cryptophytes and therefore not amplified in the oligo-dT primed cDNA synthesis.

### Nuclear-encoded plastid proteins in *D. acuminata *and their evolutionary origins

**Figure 2:**
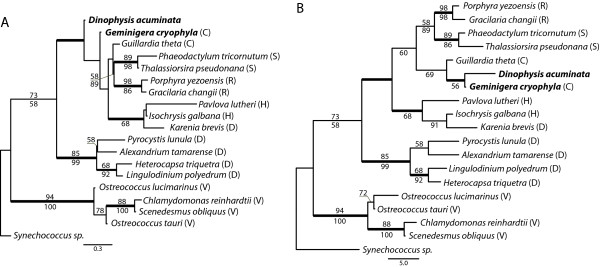
**Protein trees of *D. acuminata *psbM**. Phylogenetic trees of photosystem II subunit M protein. A) Maximum likelihood tree inferred using RAxML. B) Neighbour-joining tree inferred using PAUP*. Bold line indicates ≥ 0.95 Bayesian posterior probability for that branch. Numbers above and below branches represent bootstrap values > 50 from maximum likelihood and distance analyses, respectively. Letters in parentheses to the right of species names represent protist lineages: C, cryptophyte; D, dinoflagellate; H, haptophyte; R, red algae; S, stramenopile; V, Viridiplantae (green algae and land plants).

Of the five nuclear-encoded, plastid proteins identified in *D. acuminata*, only photosystem II subunit M appears to be of cryptophyte origin. The psbM protein is a low molecular mass subunit (33-38 amino acids on average) thought to be involved in photosystem dimer formation [33]. Due to the short length of the alignment, the maximum likelihood phylogenetic analysis of this protein was inconclusive (Figure 2a). However, the Bayesian analysis supports the grouping of *D. acuminata *and *G. cryophila*, and the neighbour-joining analysis weakly supports grouping *D. acuminata *with crytophytes (Figure 2b). The C-terminal end of psbM was also highly similar to the cryptophyte homologs. This region was not included in phylogenetic analyses because of poor conservation among algal groups (for alignment see additional File 2).

**Figure 3:**
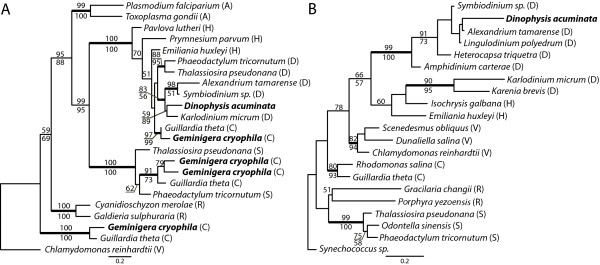
**Protein ML trees of *D. acuminata *A) TPT and B) ferredoxin**. Letters in parentheses to the right of species names represent protist lineages: A, Apicomplexa, C, cryptophyte; D, dinoflagellate; H, haptophyte; R, red algae; S, stramenopile; V, Viridiplantae (green algae and land plants). Support values for branches are indicated as in Figure 2.

Two of the plastid-related proteins, ferredoxin and the triose-phosphate transporter (TPT), group with peridinin dinoflagellates (i.e., containing the ancestral dinoflagellate plastid characterized by the photopigment peridinin) in phylogenetic analyses. These genes have either been retained from a peridinin-containing ancestor of *Dinophysis *or have been acquired from these dinoflagellates through gene transfer (Figure 3). The plastid TPT is involved in transport of fixed carbon out of the plastid [34]. This protein may provide the mechanism by which *D. acuminata *benefits from the photosynthesis occurring within the plastid by exporting the products of the Calvin cycle (e.g., glyceraldehyde-3-phosphate) to the cytoplasm. Plastid ferredoxin (petF) is the second dinoflagellate-derived plastid protein in *D. acuminata *and is responsible for distributing the electrons generated by photosystem I to various reactions in the plastid stroma [35]. The *pet*F gene is encoded on the plastid genome in cryptophytes, and a copy is presumably present in the cryptophyte plastid genome of *Dinophysis *[36], however plastid gene transcripts are not polyadenylated and therefore are not amplified in oligo dT cDNA synthesis. Although the *G. cryophila *plastid *pet*F sequence is unknown, the nuclear-encoded copy in *D. acuminata *is clearly distinct from *pet*F in the cryptophytes *G. theta *and *Rhodomonas salina *in our tree (Figure 3b). In addition, the *D. acuminata pet*F cDNA sequence contains a 5' spliced leader, a 3' UTR, and poly (A) tail strongly suggesting it is expressed from the nucleus and not the organelle.

**Figure 4:**
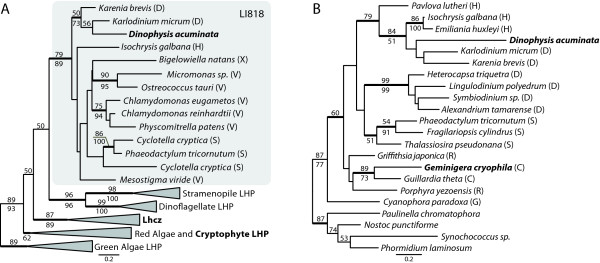
**Protein ML trees of *D. acuminata *A) LHPs and B) psbU**. Major clades of LHPs have been reduced for clarity, and clades containing *G. cryophila *representative(s) are in bold type. See additional File 3 for the full tree. Letters in parentheses to the right of species names represent protist lineages: C, cryptophyte; D, dinoflagellate; G, glaucophyte, H, haptophyte; R, red algae; S, stramenopile; V, Viridiplantae (green algae and land plants); X, Chlorarachniophytes. Support values for branches are indicated as in Figure 2.

The remaining two proteins, a light harvesting protein (LHP) and psbU, appear to be derived from either haptophytes or fucoxanthin dinoflagellates (i.e., dinoflagellates that have replaced the peridinin plastid with one derived from haptophytes and containing the photopigment fucoxanthin). LHPs shuttle the light energy captured by chlorophyll and accessory pigments to the photosystems, and algal groups have different LHPs depending on their combination of chlorophyll and accessory pigments [37]. We identified only one LHP in *D. acuminata*, a member of the distinct LI818 LHP family involved in stabilizing the photosystem in response to heat or photodamage [38-40] and may, in some situations, act as a substitute for other LHPs [39]. *D. acuminata *LI818 weakly groups with homologues from fucoxanthin dinoflagellates, *Karlodinium micrum *and *Karenia brevis *(Figure 4a). However, despite large EST datasets for cryptophytes and peridinin dinoflagellates, an LI818 family member has yet to be found in either of these groups of organisms, excluding them as a source of the LI818 gene in *D. acuminata*. Transcriptome sequencing in *G. cryophila *produced nine different LHPs, all of which grouped with other cryptophyte or red algal homologs within the Lhcz and Lhcc protein families (for the phylogenetic tree see additional File 3).

The last nuclear-encoded plastid protein identified in *D. acuminata *is the photosystem II protein psbU. Phylogenetic analyses moderately support the grouping of this protein with haptophytes and the dinoflagellates *K. micrum *and *K. brevis *(Figure 4b). PsbU, along with psbO and psbV, is extrinsically associated with photosystem II on the luminal side of the complex and enhances the oxygen evolution activity and structural stability of the complex [41,42]. PsbU is specifically involved in protecting the photosystem from heat and photodamage and may have an increased functional interaction with photosystem II when PsbO is absent [43]. PsbO is nuclear-encoded in eukaryotes and thus far missing from the *D. acuminata *transcriptome dataset.

*Dinophysis *and *Karenia/Karlodinium *are not considered close relatives; therefore, it is likely that the genes encoding LI818 and psbU were acquired through horizontal gene transfer (HGT) [44]. Interestingly, another member of this genus, *Dinophysis mitra*, is reported to have haptophyte-like plastids, suggesting that these genes could have been acquired during an earlier association with a haptophyte in the ancestor of *D. acuminata *[45]. HGT of plastid-related genes has been shown to be widespread in chromalveolates, particularly in heterotrophic taxa [46,47].

### Targeting peptides

If *D. acuminata *nuclear-encoded proteins function in the cryptophyte plastid, they should contain targeting peptides that facilitate their import into the organelle. Primary plastids, such as those in red algae and land plants, are surrounded by two membranes, and these proteins require an N-terminal transit peptide for plastid import. Proteins targeted to secondary plastids with four (e.g., cryptophytes, haptophytes) or three membranes (e.g., peridinin dinoflagellates) possess a bipartite leader sequence composed of a signal peptide, to target the protein to the endoplasmic reticulum, followed by a plastid transit peptide [48-50]. Phylogenetic analyses show that the plastid proteins of *D. acuminata *are derived from algae with three- or four-membrane bound plastids, suggesting they ancestrally contained both signal peptide and transit peptide. However, only two membranes surround the plastid in *D. acuminata*. Therefore, we expect the targeting peptides of plastid genes in *D. acuminata *to resemble the transit peptides found in organisms with primary plastids.

All of the plastid genes in *D. acuminata *possess putative transit peptides (for sequences see additional File 4). Only ferredoxin is predicted to contain a bipartite leader composed of both a signal peptide and transit peptide. The other four genes have simple transit peptides as predicted by plastid ultrastructure. PsbU, a protein that functions within the plastid lumen, also contains a twin-arginine signal peptide that directs it through the twin-arginine translocase into the plastid lumen [51]. ChloroP predicted transit peptide cleavage sites for all five proteins, but only scored those of psbM and psbU as statistically significant (score > 0.5). WoLF PSORT, a second tool for predicting protein subcellular localization, classified all five *D. acuminata *proteins as plastid-targeted. The putative transit peptide of ferredoxin contains a phenylalanine motif that is found in red algae and chromalveolates [49,52]. Plastid membrane proteins like TPT possess transit peptides structurally different from those for proteins targeted to the stroma or thylakoid membrane and therefore are not identified by programs like ChloroP [53].

### The plastids of *Dinophysis*: plastids in transition?

The discovery of plastid-targeted TPT and ferredoxin reveals a link between the metabolism of *Dinophysis *and its cryptophyte plastid. Presumably, endogenous metabolite transporters would be present in the plastid membrane of newly-acquired plastids, but having a nuclear-encoded transporter protein may allow *Dinophysis *to extend the useful life of the plastid by replacing damaged proteins. Likewise, proteins involved in stabilization of the photosystem (LI818, psbU, psbM) also may explain why the plastid can be maintained for a long period of time by protecting components of the photosystem.

**Figure 5:**
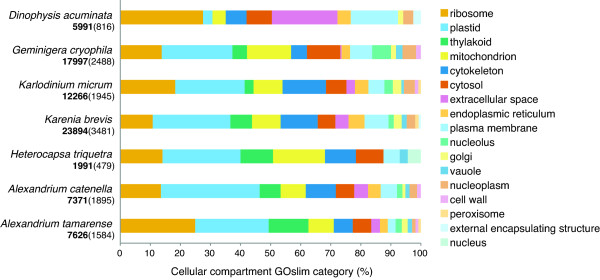
**Percent cellular compartment GOslim terms in *D. acuminata *compared to *G. cryophila *and five photosynthetic dinoflagellates**. For each species, the amount of cellular compartment GOslim terms is expressed as a percentage of the total number of unigenes annotated. The total number of unigenes used in the comparative analysis is in bold. The number of unigenes annotated by Blast2GO is in parentheses.

Although this study has found that *Dinophysis *does have nuclear-encoded genes that presumably function in the plastid, the results are not consistent with this organism having the ability to permanently maintain the plastid with its native gene complement. We identified far fewer nuclear-encoded plastid genes (only 16 by GO annotation and only 5 when highly curated) than are typically found when sequencing the transcriptome of a truly autotrophic alga (Figure 5). In addition, unlike the transcriptomes of dinoflagellates with permanent plastid replacements, only one gene of the five identified potentially originated from the source of the plastid. In *K. brevis *and *K. micrum*, dinoflagellates that have plastids derived from haptophytes, a large proportion of the nuclear-encoded plastid genes were derived from the plastid donor [54,55]. Although our unigene dataset for *Dinophysis *is not comprehensive, the results of cDNA sequencing from dinoflagellates and other algae indicate that plastid related genes are highly expressed in algae, and we would expect to have discovered many more plastid genes if *Dinophysis *possessed the full complement necessary for plastid maintenance.

Early steps for establishing a permanent organelle may have occurred in *D. acuminata*, including the development of a mechanism for metabolite exchange under host control, however, it may be difficult for another critical step in plastid acquisition, massive transfer of genes from the endosymbiont to host nucleus, to happen in *Dinophysis*. Not only is the plastid not acquired directly from the cryptophyte donor, but also the cryptophyte nucleus and nucleomorph are not retained. Genes encoded on these genomes would be needed to establish a permanent organelle, but because of the indirect method of plastid capture, *Dinophysis *may not experience the frequent exposure to the cryptophyte genomes that would be necessary for large-scale gene transfer. It is possible that *Dinophysis *could acquire the necessary genes from other sources, as they have done with four of the genes that we identified. However, this would appear to be more difficult than obtaining a co-evolved set of genes from a single source. The indirect mode of plastid acquisition in *D. acuminata *may ultimately be a barrier to establishment of a permanent plastid.

## Conclusions

The transcriptome analysis of *D. acuminata *has identified five nuclear-encoded plastid genes that appear to be targeted to the dinoflagellate plastid and are derived from multiple algal lineages. Only *psb*M appears to be derived from a cryptophyte. Ferredoxin and TPT group with peridinin dinoflagellates and have either been retained from a peridinin plastid-containing ancestor or acquired through HGT. The other genes (LI818 and *psb*U) are derived from either fucoxanthin dinoflagellates (*Karenia/Karlodinium*) or haptophytes. The light harvesting protein, LI818, and the two photosystem II subunits appear to be involved in stabilizing and protecting the photosystem, while ferredoxin and TPT function in exporting the products of photosynthesis from the plastid. These findings suggest that *D. acuminata *has some functional control of its plastid, but the minimal amount of plastid-related genes compared to other fully phototrophic algae suggests that *D. acuminata *does not have the ability to permanently maintain the plastid.

## Methods

### Cultures

*D. acuminata *strain DAEP01 was established from Eel Pond, Woods Hole, MA in September of 2006. Cells were cultured using the two step feeding system described by Park et al. [56] where *D. acuminata *(DAEP01) is fed the ciliate, *M. rubra *(CCMP2563), which is fed the cryptophyte, *G. cryophila *(CCMP2564). The three algae are cultured in modified f/2-Si medium [57] at 4°C on a 14:10 h L:D cycle. *G. cryophila *is fed to *M. rubra *upon reaching a cell density of 500,000 cells mL^-1 ^(2 mL of *G. cryophila *is added to 3 mL M. rubra in 20 mL f/2 medium). Once *M. rubra *cultures are completely clear of *G. cryophila*, the ciliate is fed to *D. acuminata *(3 mL *M. rubra *at ~14,000 cells mL^-1 ^is added to 2 mL *D. acuminata *at ~1800 cells mL^-1 ^in 20 mL f/2 medium). Weekly cell counts of *D. acuminata *fixed in Utermöhls solution [58] were performed at 100× magnification in a Sedgewick rafter counting chamber. Additional *M. rubra *cells were added to *D. acuminata *cultures when the *Myrionecta/Dinophysis *cell ratio fell below 1:1. Cultures of *D. acuminata *were inspected for the presence of *M. rubra *prey by light microscopy and harvested for RNA extraction at least one week after *M. rubra *was no longer observed in the cultures.

### RNA extraction and cDNA synthesis

The dinoflagellate specific cDNA synthesis and amplification was performed using the Clontech Super SMART PCR cDNA Synthesis Kit. The first-strand synthesis reaction included 1 μg of total RNA and 84 pmoles of modified 3' SMART CDS Primer IIA (5' AAG CAG TGG TAT CAA CGC AGA GTT TGT TTT TTT TTC TTT TTT TTT TVN 3'). The reaction was incubated at 42°C for 90 min. The first-strand cDNA was column purified using the Clontech NucleoSpin Extract II Kit according to Super SMART cDNA synthesis protocol. The cDNA amplification was performed using the Clontech Advantage 2 PCR kit. The amplification reaction included 40 μl of purified first-strand cDNA, 20 pmoles 5' trans-spliced leader primer (5' TCC GTA GCC ATT TTG GCT CAA G 3'), and 20 pmoles PIIA PCR primer (5' AAG CAG TGG TAT CAA CGC AGA GT 3'). Cycling parameters included an initial denaturation step at 95°C for 1 min followed by 26-29 cycles of 95°C for 15 sec, 65°C for 30 sec, 68°C for 6 min. PCR products were visualized on an agarose gel to confirm expected cDNA size range and cleaned using the Clontech CHROMA SPIN -1000 size selection columns.

First strand synthesis of *M. rubra *and *G. cryophila *cDNA was performed using the Invitrogen Superscript III First-Strand Synthesis System. The first-strand reaction included 1 μg of total RNA and 50 pmoles modified oligo dT primer with PIIA tag (see above). The reaction was incubated at 50°C for 90 min. PCR amplification of the first-strand cDNA was performed using the Clontech Advantage 2 PCR kit. The reaction included 10 μl of first-strand reaction and 10 pmoles PIIA PCR primer (5' AAG CAG TGG TAT CAA CGC AGA GT 3'). Cycling parameters included an initial denaturation step at 95°C for 1 min followed by 18 cycles of 95°C for 30 sec, 58°C for 30 sec, 68°C for 6 min. PCR products were visualized on an agarose gel to confirm expected cDNA size range and cleaned using the Clontech CHROMA SPIN -400 size selection columns.

### Sequencing, PCR and cloning

The cDNA was sequenced with a 454 FLX pyrosequencing machine at the Arizona Genomics Institute (Tucson, AZ, USA) and data was assembled with SeqMan (DNASTAR, Madison, WI, USA) or gsAssembler (Roche NimbleGen, Inc., Madison, WI, USA). *D. acuminata *contigs were queried against the *M. rubra *and *G. cryophila *assemblies using BLASTN to determine if sequences from these species were present in the *D. acuminata *dataset. Contigs were annotated using Blast2GO [59], and *D. acuminata *plastid gene fragments were identified using those annotations as well as BLASTX searches against the non-redundant protein database at NCBI. Full-length transcripts were independently verified by PCR from new and unamplified *D. acuminata *cDNA generated using the SuperScript III First-Strand Synthesis System (Invitrogen Co., Carlsbad, CA, USA). Gene specific internal primers were designed from 454 sequence fragments and paired with either the 5' trans-spliced leader primer or an anchored oligo dT primer for PCR amplification of the 5' and 3' ends, respectively. PCR products were sequenced directly or cloned into pGEM-T Easy vectors (Promega, Madison, WI, USA) and sequenced using BigDye dye terminator sequencing (Applied Biosystems, FosterCity, CA USA) on an automated DNA sequencer (ABI 3730 × l, Applied Biosystems). *D. acuminata *plastid protein sequences were searched against the *G. cryophila *transcriptome assembly using TBLASTN to identify *G. cryophila *homologs. The plastid cDNA sequences have been deposited in Genbank (Accession numbers HM125141-HM125145).

### Phylogenetic analysis and targeting peptide determination

Amino acid sequences of *D. acuminata *and *G. cryophila *were aligned with algal sequences from Genbank using ProbCons with default parameter settings [60]. Gblocks was used to remove poorly aligned regions of the alignments [61]. Distance analyses were performed in PAUP* v4.0b10 [62] with 100 bootstrap replicates using a neighbor-joining search with minimum evolution as the objective function and uncorrected distances. The best-fit model for each alignment was identified by ProtTest v1.4 [63] using the AIC model selection criterion and a BIONJ tree. The ProtTest best-fit evolutionary model for each data set was applied to the maximum likelihood (ML) and Bayesian analyses. ML trees were inferred using the Cipres web portal RAxML rapid bootstrapping and ML search [64,65]. Bayesian analyses were performed in BEAST v1.4.7 [66] assuming an uncorrelated lognormal relaxed molecular clock model using the substitution and site heterogeneity models determined by ProtTest and with a Yule process speciation tree prior. Two independent runs (5 million - 10 million steps) were performed for each analysis and terminated once examination of the Markov chain Monte Carlo (MCMC) samples in Tracer v1.4.1 http://beast.bio.ed.ac.uk/Tracer indicated convergence of the chains with estimated sample sizes greater than 200. The maximum clade credibility tree topology was determined from resulting MCMC tree samples using TreeAnnotator v1.4.7 [66]. Amino acid sequences were screened for targeting peptides and associated cleavage sites using SignalP v3.0, ChloroP v1.1, TatP v1.0, and Wolf PSORT [67].

## Authors' contributions

JHW and JDH designed and performed the research, analyzed the data and wrote the manuscript.

## Supplementary Material

Additional file 1 **Table of all *D. acuminata *contigs called by Blast2GO**. Blast2GO analysis identified 16 contigs as potentially plastid-related based on a cellular compartment GOslim category of plastid or thylakoid. Click here for file

Additional file 2 **Protein alignment of psbM**. Alignment to the left of the black line was used for phylogenetic analyses. The C-terminal ends to the right of the line were trimmed by Gblocks due to poor sequence alignment. The C-terminal ends of *Guillardia theta*, *G. cryophila*, and *D. acuminata *are outlined in black. Click here for file

Additional file 3 **Full protein ML tree of plastid light harvesting proteins (LHP)**. Trees were inferred using RAxML. Bold line indicates ≥ 0.95 Bayesian posterior probability for that branch. Numbers above and below branches represent bootstrap values > 50 from maximum likelihood and distance analyses, respectively. Click here for file

Additional file 4 **Targeting peptides of *D. acuminata***. The petF (ferredoxin) peptide has both a putative signal and transit peptide. Only psbU possesses a twin-arginine signaling peptide. An N-terminal phenylalanine transit motif, found in red algae and chromalveolates, was detected in ferredoxin. Click here for file
