# Supplementary material for: Transcriptome analysis reveals nuclear-encoded proteins for the maintenance of temporary plastids in the dinoflagellate Dinophysis acuminata
Source: BMC Genomics. 2010 Jun 10;11:366. doi: 10.1186/1471-2164-11-366 (PMC3017763; doi:10.1186/1471-2164-11-366)
Supplement: Additional file 1 — Table of all D. acuminata contigs called by Blast2GO. Blast2GO analysis identified 16 contigs as potentially plastid-related based on a cellular compartment GOslim category of plastid or thylakoid. [file 1471-2164-11-366-S1.PDF]

Additional file 1: Blast2GO analysis identified 16 contigs as potentially plastid-related based on a cellular compartment GOslim term of plastid or thylakoid

Contig	Blast2GO suggested annotation	Contig length	Blast results		Gene Ontology results	
			eValue	mean similarity	#GO terms	GOs
116	phosphoribosylformimino-5-aminoimidazole carboxamide ribotide isomerase	273	1.33E-17	72.05%	4	F:catalytic activity; C:plastid; P:biosynthetic process; P:cellular amino acid and derivative metabolic process
1419	serologically defined breast cancer antigen 84	318	1.50E-25	63.05%	2	C:mitochondrion; C:plastid
198	photosystem i reaction center subunit iv	220	1.45E-19	85.70%	7	C:thylakoid; C:cytoplasm; C:plasma membrane; C:membrane; C:plastid; F:catalytic activity; P:photosynthesis
4042	chloroplast ferredoxin	155	7.47E-08	93.35%	6	P:protein metabolic process; P:cellular process; F:binding; F:molecular_function; P:transport; C:plastid
4120	zinc an1-type domain 2a	193	1.36E-09	65.65%	1	C:plastid
5703	photosystem ii oxygen evolving complex protein	689	1.11E-09	55.80%	1	C:thylakoid
5767	chloroplast light harvesting protein isoform 4	911	8.19E-21	61.90%	4	P:generation of precursor metabolites and energy; P:photosynthesis; C:plastid; C:membrane
5893	60s ribosomal protein l23a	658	7.44E-37	80.35%	7	C:ribosome; F:structural molecule activity; P:translation; F:RNA binding; C:plastid; F:nucleotide binding; P:cellular process
6038	sec61-gamma subunit of protein translocation	508	2.36E-23	78.85%	7	P:transport; P:cellular process; C:membrane; C:endoplasmic reticulum; F:protein binding; F:transporter activity; C:plastid
6044	sec61-gamma subunit of protein translocation	433	7.30E-24	78.35%	7	P:transport; P:cellular process; C:membrane; C:endoplasmic reticulum; F:protein binding; F:transporter activity; C:plastid
6217	retrotransposon unclassified	264	1.31E-44	100.00%	2	C:mitochondrion; C:plastid
6270	transposon expressed	223	7.16E-35	79.35%	1	C:plastid
6342	hgwp repeat containing	241	2.29E-25	61.75%	1	C:plastid
6349	cysteinyl-trna expressed	230	1.07E-14	78.70%	1	C:plastid
718	actin-binding protein	224	7.76E-05	64.30%	1	C:plastid
889	clathrin coat assembly protein	507	6.42E-35	66.25%	6	C:membrane; C:cytoplasm; P:transport; P:cellular process; C:plastid; F:protein binding
