# Supplementary material for: Transcriptome analysis reveals nuclear-encoded proteins for the maintenance of temporary plastids in the dinoflagellate Dinophysis acuminata
Source: BMC Genomics. 2010 Jun 10;11:366. doi: 10.1186/1471-2164-11-366 (PMC3017763; doi:10.1186/1471-2164-11-366)
Supplement: Additional file 2 — Protein alignment of psbM. Alignment to the left of the black line was used for phylogenetic analyses. The C-terminal ends to the right of the line were trimmed by Gblocks due to poor sequence alignment. The C-terminal ends of Guillardia theta, G. cryophila, and D. acuminata are outlined in black. [file 1471-2164-11-366-S2.PDF]

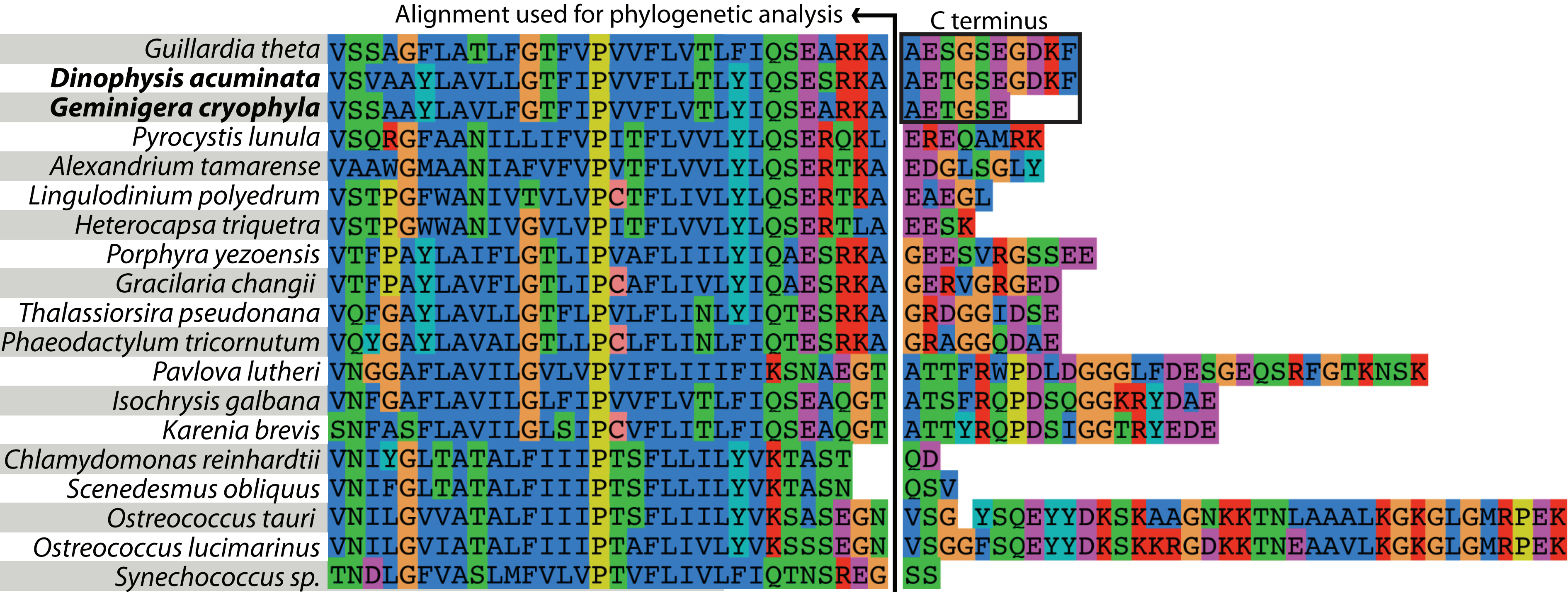

Alignment used for phylogenetic analysis
C terminus
Guillardia theta VSSAGFLATLFGTFVPVVFLVTLFIQSEARKA AESGSEGDKF
Dinophysis acuminata VSVAAYLAVLLGTFIPVVFLLTLYIQSESRKA AETGSEGDKF
Geminigera cryophyla VSSAAYLAVLFGTFIPVVFLVTLYIQSEARKA AETGSE
Pyrocystis lunula VSQRGFAANILLIFVPITFLVVLYLQSERQKL EREQAMRK
Alexandrium tamarense VAAWGMAANIAFVFVPVTFLVVLYLQSERTKA EDGLSGLY
Lingulodinium polyedrum VSTPGFWANIVTVLVPCTFLIVLYLQSERTKA EAEGL
Heterocapsa triquetra VSTPGWWANIVGVLVPITFLVVLYLQSERTLA EESK
Porphyra yezoensis VTFPAYLAIFLGTLIPVAFLIILYIQAESRKA GEESVRGSSEE
Gracilaria changii VTFPAYLAVFLGTLIPCAFLIVLYIQAESRKA GERVGRGED
Thalassiorsira pseudonana VQFGAYLAVLLGTFLPVLFLINLYIQTESRKA GRDGGIDSE
Phaeodactylum tricornutum VQYGAYLAVALGTLLPCLFLINLFIQTESRKA GRAGGQDAE
Pavlova lutheri VNGGAFLAVILGVLVPVIFLIIIFIKSNAEGT ATTFRWPDLDGGGLFDESGEQSRFGTKNSK
Isochrysis galbana VNFGAFLAVILGLFIPVVFLVTLFIQSEAQGT ATSFRQPDSQGGKRYDAE
Karenia brevis SNFASFLAVILGLSIPCVFLITLFIQSEAQGT ATTYRQPDSIGGTRYEDE
Chlamydomonas reinhardtii VNIYGLTATALFIIIPTSFLLILYVKTAST QD
Scenedesmus obliquus VNIFGLTATALFIIIPTSFLLILYVKTASN QSV
Ostreococcus tauri VNILGVVATALFIIIPTSFLIILYVKSASEGN VSG YSQEYYDKSKAAGNKKTNLAAALKGKGLGMRPEK
Ostreococcus lucimarinus VNILGVIATALFIIIPTAFLIVLYVKSSSEGN VSGGFSQEYYDKSKKRGDKKTNEAAVLKGKGLGMRPEK
Synechococcus sp. TNDLGFVASLMFVLVPTVFLIVLFIQTNSREG SS
