# Supplementary figures and images for: Transcriptome analysis reveals nuclear-encoded proteins for the maintenance of temporary plastids in the dinoflagellate Dinophysis acuminata
Source: BMC Genomics. 2010 Jun 10;11:366. doi: 10.1186/1471-2164-11-366 (PMC3017763; doi:10.1186/1471-2164-11-366)

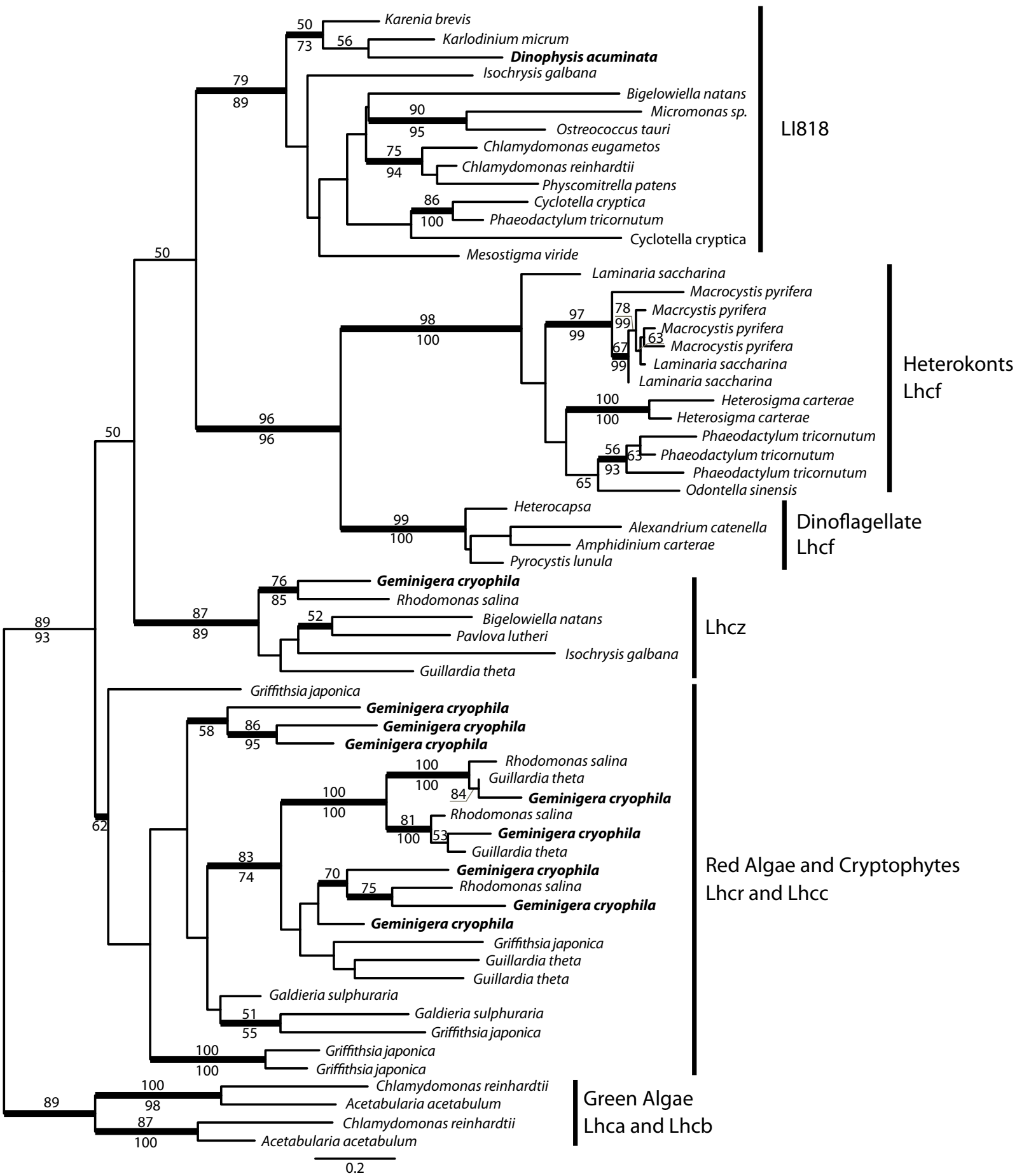

Supplement: Additional file 3 — Full protein ML tree of plastid light harvesting proteins (LHP). Trees were inferred using RAxML. Bold line indicates ≥ 0.95 Bayesian posterior probability for that branch. Numbers above and below branches represent bootstrap values > 50 from maximum likelihood and distance analyses, respectively. [file 1471-2164-11-366-S3.PDF]
