# Supplementary material for: Transcriptome analysis reveals nuclear-encoded proteins for the maintenance of temporary plastids in the dinoflagellate Dinophysis acuminata
Source: BMC Genomics. 2010 Jun 10;11:366. doi: 10.1186/1471-2164-11-366 (PMC3017763; doi:10.1186/1471-2164-11-366)
Supplement: Additional file 4 — Targeting peptides of D. acuminata. The petF (ferredoxin) peptide has both a putative signal and transit peptide. Only psbU possesses a twin-arginine signaling peptide. An N-terminal phenylalanine transit motif, found in red algae and chromalveolates, was detected in ferredoxin. [file 1471-2164-11-366-S4.PDF]

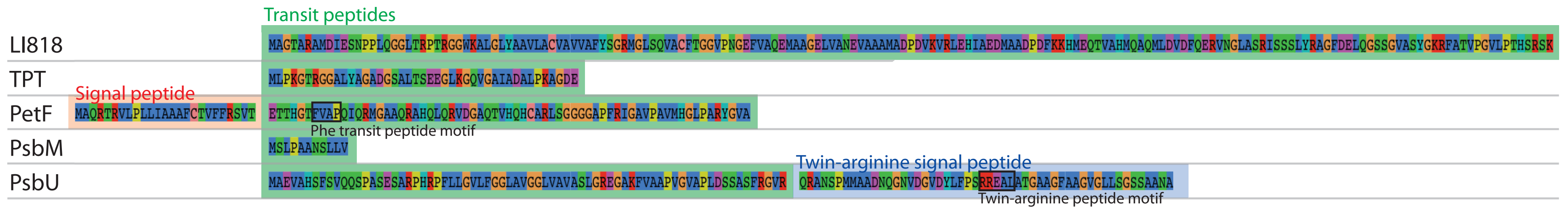
Transit peptides
LI818
MAGTARAMDIESNPPLQGGLTRPTRGGWKALGLYAAVLACVAVVAFYSGRMGLSQVACFTGGVPNGEFVAQEMAAGELVANEVAAAMADPDVKVRLEHIAEDMAADPDFKKHMEQTVAHMQAQMLDVDFQERVNGLASRISSSLYRAGFDELQGSSGVASYGKRFATVPGVLPTHSRSK
TPT
MLPKGTRGGALYAGADGSALTSEEGLKGQVGAIADALPKAGDE
Signal peptide
PetF
MAQRTRVLPLLIAAAFCTVFFRSVT
ETTHGTFVAPQIQRMGAAQRAHQLQRVDGAQTVHQHCARLSGGGGAPFRIGAVPAVMHGLPARYGVA
Phe transit peptide motif
PsbM
MSLPAANSLLV
Twin-arginine signal peptide
PsbU
MAEVAHSFSVQQSPASESARPHRPFLLGVLFGGLAVGGLVAVASLGREGAKFVAAPVGVAPLDSSASFRGVR
QRANSPMMAADNQGNVDGVDYLFPSRREALATGAAGFAAGVGLLSGSSAANA
Twin-arginine peptide motif
